# Top-down internal communication and its importance for the sustainability of agricultural organizations from the perspective of Tomas Bata´s management philosophy

**DOI:** 10.1371/journal.pone.0291087

**Published:** 2023-09-19

**Authors:** Hana Urbancová, Pavla Vrabcová, Zuzana Pacáková, Šárka Janků

**Affiliations:** 1 Department of Human Resources, University of Economics and Management, Prague, Czech Republic; 2 Department of Economic Statistics, Faculty of Economics, Technical University of Liberec, Liberec, Czech Republic; 3 Department of Statistics, Faculty of Economics and Management, Czech University of Life Sciences, Prague, Czech Republic; 4 Department of Management, University of Economics and Management, Prague, Czech Republic; University of Naples Federico II: Universita degli Studi di Napoli Federico II, ITALY

## Abstract

The benefits of internal communication not only can be associated with higher performance, but also with increased awareness of the values within an organization, setting and achieving objectives, getting involved in beneficial activities, encouraging employees’ personal development and, last but not least, educating and motivating employees to take responsibility. A responsible working environment facilitates effective internal communication, which is in line with the principles of the Bata Management System. The aim of this paper is to evaluate whether setting top-down internal communication is in line with the sustainable principles of Tomas Bata in organizations in the Czech Republic. The article assesses workplace communication and relationships, including social sustainability and social responsibility, focusing on their added value for corporate practice in all areas of business. The study is based on quantitative research in organizations across sectors and qualitative research in agricultural organizations (questionnaire survey *n*_*1*_ = 183; focus group *n*_*3*_ = 5), using the tools of descriptive statistics, logistic regression and cluster analysis. The results have shown that combining methods of top-down communication is crucial for communicating information efficiently, stimulating employees and achieving the organization´s objectives and that Tomas Bata´s philosophy concerning communication setting can be implemented even more easily in the current digital age than it was in the past if organizations are interested. The research contributes to a better understanding of how the Bata Management System can be applied within each organization, how effective communication settings will help prevent conflicts that workers are engaged in, increase their stabilization, and facilitate the application of Bata’s sustainable legacy in the international space. Research makes a significant contribution to advancing knowledge of effective communication from the perspective of Bata for the sustainability of agricultural organizations where the principles have not yet been explored. This is evidenced by the zero overlap with previously published articles despite the rapid progress of research and technology.

## Introduction

During these turbulent times, the strategic focus of organizations across sectors is changing and it is impossible to adopt and implement the exact strategies that were successful in the past [1]. It is necessary to always look for principles that create synergy and have a positive effect in different organizations at different points in time. This is met by the Bata Management System [2], which is found in modern management methods, economic systems, personnel approaches, or process optimization, and especially in educating and training less experienced workers. One can observe that, although the concept of the Bata Management System is being abandoned, even today, the integration of positive attitudes, good sustainable practices, and programmes developing business strategy are constantly used and adapted to current conditions [3] including the workplace welfare of employees [4].

The principles and philosophy of Tomas Bata are natural as they are based on common sense. Tomas Bata frequently stated, *“I didn´t build a factory*. *I developed the people and then they built the factory*.” He knew that the success of an organization is built on its employees and their skill levels, as evidenced by another one of his quotes: “*No business will ever become great unless it finds a way to change laborers into directors*.” This approach distinguishes a modern dynamic organization from a rigid one that does not follow the current trends [5]. Creating quality conditions for employees to do their work is directly dependent on the quality of the entire organization. A quality working environment is not only about premises, technologies, or materials [6], but primarily about internal communication [7]. If management fails to communicate with their employees, provide quality information and communicate the organization´s goals and visions [8], they cannot expect loyalty and motivation for better performance from their employees. Therein lies “Bata´s” secret of success. Tomas Bata did not have employees; rather, he always had co-workers [5]. Of course, such a philosophy of internal communication, in which employees feel safe and free to express themselves and state their opinions, must be set by a management team aiming to achieve a sustainable business.

One of the most important and pervasive elements for promoting sustainability in organizations, regardless of the sector (primary, secondary, or tertiary), is internal sustainability communication [9] and its setting [10] including the use of innovation in communication [11].

Maintaining and developing relationships in the workplace and among team members is a trend not only in human resource management. Building relationships among all employees, fostering the maintenance and development of a positive individual [1], corporate culture [12] and teamwork relationships [13] are important factors for cohesion and deepening the intention of individuals to remain in an organization that is based on good relationships and a sense of belonging.

Most organizations in all sectors of the economy in current corporate practice emphasize sustainability in their goals, a principle that supports the Bata management system. This principle is newly supported to achieve a sustainable system of agriculture in the EU (Common Agricultural Policy), which combines the social, economic and environmental approaches of agricultural organizations. A primarily social approach supports the development of agriculture and forestry towards the fulfilment of essential tasks for the benefit of the wider society and helps achieve economic goals and support environmental goals. This is not possible without effective communication between all interest groups and also in individual agricultural and forestry organizations. The entire philosophy of Bata is interwoven with the need to set up effective communication in the organization. Subsequently, it is possible to effectively fulfil the goals and move towards sustainability.

Building positive working relationships is based on effective communication [14], which creates the right preconditions for enhancing employee motivation to work well [1] and perform better [15]. An important factor in employee satisfaction is the organizational climate and effective communication among employees [16], as there is an increase in job performance among them despite poorer working conditions. The development of more complex working conditions has been instigated not only by the COVID-19 pandemic but also by the war in Europe and the resulting negative economic consequences for the EU as a whole. Although it was introduced around the world in the first half of the last century, as far as we know, there has been no research as of yet integrating the principles of the Bata Management System and the setting of top-down internal communication management.

Related to this is the transparency of communication and the regular publication of sustainability reports to all employees in agricultural organizations. Increasing the awareness and adaptability of employees to sustainability and creating an effective and transparent system for sharing information and knowledge. This will help promote the perception of sustainable development as a natural part of the daily life of an agricultural organization. Within the internal communication plan with employees, it is advisable to include topics from the field of sustainability.

The knowledge gap is in analysing the present status of internal communication settings in organizations in the Czech Republic in accordance with the principles of the Bata Management System, including agricultural organizations.

This paper aims to evaluate whether the methods of setting top-down internal communication align with Tomas Bata´s sustainable principles in organizations in the Czech Republic. According to Tomas Bata, the art of top-down communication is primarily an innate ability, which is closely connected with self-confidence, humility, and the ability to listen. The ability to let others speak and the ability to make decisions and stand behind one´s decisions are absolute prerequisites for proper top-down communication. A manager who can communicate Tomas Bata´s philosophy downwards, towards his or her co-workers, is a true leader, whom people stand by and follow when fulfilling the set visions and objectives of the organization. Research question 1 (hereafter RQ 1): What types of top-down communication and their combination are effective for ensuring sustainable communication in accordance with the Bata Management System in organizations?

The article presents ways of achieving effective managing of communication in agricultural organizations according to the application of Bata principles with focus on internal communication, which was not and is not used for this sector. A change in thinking and the application of principles with emphasis on communication in agricultural organizations will help the development of the field and the solution of managerial problems caused by ineffective communication causing conflicts or problems in following processes.

## Theoretical background

In accordance with the Bata Management System for the cohesion of teams and work groups, not only education, abilities, skills, and experience, but also, primarily, the ability to be part of a team on the basis of synergy, equality, and loyalty to successfully build relationships leading to cooperation and increasing the performance of both the individual as well as the entire team needs to be emphasized [17].

Tomas Bata was a brilliant strategist who based his success mainly on people and communicating with them [18]. By being able to listen to his co-workers, he could expand his empire in a meaningful way. This is one of the reasons why he founded, in conjunction with industrial fields, agricultural fields. There is a logical explanation for the development of agricultural production, which is seemingly unrelated to the shoe business. Tomas Bata assumed that along with the development of industry, it would also be possible to develop agriculture, which would supply the industry with quality, cheap, and controlled products [4]. Industry and agriculture were interlinked in an integrated way emphasizing mutual cooperation and support.

To maximize work efficiency, workers must have a quality work environment, equipment, and especially a quality diet [18]. Tomas Bata led farmers to focus on meat, dairy, fruit, and vegetable production.

Collaboration between industry and agriculture also brought other benefits. The industry provided agriculture with new machinery and technologies so that it could produce more quality food and expand its reach and scope of activity. The same rules applied to both industrial and agricultural workers, namely the quality and safe working environment, the continuous process of improvement and education, well thought-out and effective advertising, and, above all, open communication.

It is necessary to realize why the principles of Bata have not yet been implemented in agricultural organizations and communication in not a priority in agricultural organizations. It is primarily with regard to the specificity of the agricultural sector, where there are limits in fulfilling the main features of Bata management. Within agricultural organizations, direct top management is frequent, taking into account the fact that the Czech Republic has the most significant number of small agricultural organizations (up to 10 employees). The control system takes place either directly or not taking into account the insufficient number of employees in agriculture, the dependence of the performance of operational tasks on the current weather or the existence/non-existence of resources. Operability and flexibility are not as high in agricultural organizations as in organizations in other sectors. Management is not simple and often on the border of expediency, while the economic evaluation of decisions is primary in agricultural organizations. In agricultural enterprises, they are not primarily concerned with setting up communication for effective management and achieving process sustainability. While it can be said that agricultural enterprises are trying to introduce new techniques into production (SMART farming) and innovations, these are, closely connected with research and development activities, where agricultural organizations encounter personal and financial limits (e.g. lack of subsidy titles, state support etc.), at the same time they have to work with a vast range of risks.

This new, corporate philosophy of linking industry and agriculture was often mentioned by the press. Thematically focused articles and professional papers served to naturally promote and present the Bata Group´s policy as an efficient manager and provider of supplies to the city´s inhabitants. The articles also advertised the products, emphasizing their freshness, availability, low prices, or salubrity. The importance of communication and sharing information is evident not only from the above [18].

Internal communication in an organization is a tool for creating a suitable working environment and organizational climate [19]. Broadly speaking, internal communication can also include organizational culture [20] or attitudes towards the organization´s environmental impact [21] and its support of Corporate Social Responsibility (CSR) [22]), where all employees and managers constitute the primary target group of the organization [23]. The setting of effective internal communication is aimed at:

- providing the information necessary for performing a specific job (information related to the specific job position, the purpose of the job, the related expectations, and the relation of this job to other processes in the organization) and coordinating the job´s position and its association within the entire structure of the organization [24];
- ensuring mutual understanding and cooperation within the organization by sharing information that stimulates employees and leads to mutual understanding, developing relationships, and sharing organizational objectives [9];
- ensuring the stability of the organizational environment and the loyalty of employees by, among other things, communicating information regarding the organization´s attitudes to its current position, future developments, or planned changes with a focus on leadership [8].

The principles mentioned above, as well as the two below, are entirely based on Bata´s management philosophy and constituted a significant competitive advantage for the Bata firm [17]:

- building mutual trust in communication at all levels and communicating information based on verified facts, and
- no withholding of information, no matter how uncomfortable it is, towards employees at all levels by management.

Vertical communication between parties usually includes orders, dispositions, recommendations, advice, instructions, organizational or technical standards, work manuals, annual rights, etc. The results section evaluates the types of top-down communication in the context of the principles of the Bata Management System, see Table 1.

**Table 1:** Types of communication and their context with the principles of the Bata Management System.

Type of communication	Context with the principles of the Bata Management System	References
Brainstorming, brainwriting	World-famous management systems, e.g., Bata or Ford, were based on Taylor´s theory. Initially, business processes were documented and described for use at work, but they were fixed; there was no intentional measurement or optimization, and there was rarely any improvement. The present management systems based on the Bata Management System use creative methods to support the generation of new ideas and their development.	[5, 17, 25]
Electronic communication	Communication efficiency goes hand in hand with time-saving, and Bata also paid attention to timeliness and accuracy when communicating information.	[17, 25]
Communication through employee representatives or a trade union body	Bata founded a trade union to take care of the social needs of employees. Once the union leaders started talking about a strike, he immediately dissolved the union and said that he would take care of the employees´ welfare himself, and he did so with an effective set strategy aimed at supporting CSR.	[2, 23, 17, 26]
Online meetings, webinars, video conferences	Obviously, Bata could not use online tools at the beginning of the last century, but it can be stated that he paid attention to the technical and organizational levels leading to work discipline, cooperation, efficiency, and economy.	[17, 25, 27]
Personal team meetings, working meetings, discussions	Fair and clear top-down communication was very important to Tomas Bata. Although he did not like resistance and was very reluctant to admit his mistakes and errors, he was always fair and direct. He required this also from his managers and directors.	[17, 25, 27]
Written communication, intended directly for employees	For internal (with employees) and external (with customers and the general public) communication, Bata made effectively and targeted use of newspapers and magazines.	[3, 18, 24]
Specialized evaluation and feedback process in SW	In the context of feedback control, Bata paid attention to regular feedback from management as well as feedback from his employees. He emphasized direct communication. The appraisal and feedback processes are dealt with by sophisticated software.	[18, 24]
Oral communication intended directly for employees	Oral communication was a priority in the Bata Management System. He emphasized face-to-face meetings and direct communication to eliminate information on the grapevine or misunderstanding of the meaning of the message being communicated.	[17, 25]

Source: own processing based on the sources mentioned

In terms of communication, informal face-to-face communication, especially top-down, is a specific feature of agricultural organizations. These are especially face-to-face meetings within teams, work discussions, one-to-one meetings, and oral communication aimed directly at employees for their professional growth. In a company with a democratic style of management, communication flows are expanded to include flows that carry information from the bottom up and across the organizational structure.

Supporting the professional growth of employees was an indispensable part of Bata´s philosophy. “*Direct conduct is one of the primary conditions of success*! *If you act openly with the intention to help people*, *your success is inevitable*.” The level of setting top-down communication in the selected agricultural organizations in the Czech Republic is presented below.

In summary, some other studies focus mainly on the IT sector and project management in general [11] or focus on communication in general [21]. Only a few studies compare the situation across multiple organizations [21] or different sectors [28]. For implementation, the entire issue of a sustainable communication setup, according to the Bata system and support and setup management theory, it is important to understand the behavior of different organizations based on their size, the sector of operation or ownership [3]. These comparative analyses are lacking, emphasizing that the Bata system is sustainable and also applicable worldwide. Important for our survey is the assessment of workplace communication and relationships, including social sustainability and social responsibility, focusing on the added value for corporate practice in all areas of business. No studies focus on a wide range of organizations in a small export economy such as the economy of the Czech Republic.

Based on the above information, we can identify a knowledge gap in terms of a lack of comparison across sectors and different organizations collectively.

## Materials and methods

The quantitative data were collected through a Google Forms questionnaire, which was completed by the middle or senior management of the organizations, or in the case of smaller organizations, by the owner. The survey was conducted in 2021. A preliminary survey (*n*_*2*_ = 10) was carried out in advance to see if the questions were understandable. To avoid duplication, IP addresses were tracked and questions that showed a match were completely excluded from the survey. The survey consists of seven questions focusing on top-down internal communication, including how its setting is approached, and five identification questions (sector, size, majority owner, type and annual turnover).

A total of 850 Czech organizations were contacted (based on a random selection from the ALBERTINA database with more than 2,800,000 business entity registration records), and 183 organizations responded to the questionnaire. Organizations were approached based on random selection, 70% from the tertiary sector, 20% from the secondary sector, and 10% from the primary sector, according to the recommendations of the Czech Statistical Office, and the response rate was 21.5%. The research in the Czech Republic was carried out primarily for the reason that Tomas Bata came from Czechoslovakia and the contribution of the article seeks, among other things, to give an overview of how companies in the Czech Republic direct his managerial legacy. The questionnaire (in anonymous form) was submitted to representatives of organizations at the middle level of management or owners in online form, and only one questionnaire was filled out by each organization. The representatives were informed in the contact email (written form) that by sending a fully completed questionnaire (partially completed ones were not included in the research), they were confirming their participation in the survey and agreeing to their answers being used for the purposes of evaluating the survey. The questionnaire survey was created in accordance with ethical codes of research in the Czech Republic (Ethical framework for research, Resolution of the Government of the Czech Republic dated August 17, 2005 No. 1005, as amended). This study does not include minors and it does not report a retrospective study of medical records or archived samples. The basic identification questions in the questionnaire survey include the following variables (see Table 2).

**Table 2:** Organizations that participated in the research–basic data.

Characteristics	Categories		
The sector of the organization	**Primary**	**Secondary**	**Tertiary**
	4.5%	40.2%	55.3%
The size of the organization	**≤50**	**51–250**	**>250**
	26.8%	27.9%	45.3%
Majority ownership	**Domestic**	**Foreign**	
	45.3%	54.7%	
The type of organization	**Private**	**Public**	**Non-profit**
	86.0%	11.2%	2.8%
Annual turnover	**≤10 mil. EUR**	**11–50 mil. EUR**	**>50 mil. EUR**
	38.5%	38.0%	23.5%

Source: own survey

Dependence between the selected communication types and identification features was tested. Five identification variables were considered and their effect on the use of the selected forms of communication was assessed using logistic regression. The Backward Likelihood Ratio method was used to select the variables in the model.

The cluster analysis was applied to identify groups of organizations that tend to use the same combinations of communication types on the basis of the preferred top-down communication types. The hierarchical clustering with Ward´s method of clustering and Squared Euclidean distance was used to find clusters. Subsequently, differences in the selected characteristics between individual clusters were tested by means of the chi-square test of independence or, more precisely, the Fisher-Freeman-Halton Exact test.

The null statistical hypotheses that were tested at the 0.05% level of significance for achieving the aim of the article and research questions are:

1. H_0_: Using the top-down communication types does not depend on the size of the organization according to the number of employees.
2. H_0_: Using the top-down communication types does not depend on the type of organization in terms of majority ownership.
3. H_0_: The representation of organizations in the individual clusters is not statistically significantly different in terms of the size by the number of employees and by the majority ownership.

To verify the study results, subsequent interviews were conducted using the focus group (*n*_*3*_ = 5). These were carried out online with managers/directors of the organizations (agricultural organizations). The interviewers (the authors of the survey) asked what practices their organizations used in terms of communication and workplace relations. The focus group lasted no longer than 90 minutes. Once the subject matter of top-down internal communication was selected, the preparation and planning phase began, and then the focus group followed, of which a detailed recording was made. Following the discussion, the interviewers (the authors of the article) further inquired about the specifics of individual areas, particularly focusing on the areas that had arisen from the focus group, namely the importance of setting effective top-down internal communication in the business practice of agricultural organizations and in the Bata Management System, including its support for CSR and business sustainability. The IBM SPSS Statistics 28 statistical software was used to evaluate the results. The condition of a minimum number of respondents in this research (*n* = 164) was satisfied in accordance with Krejcie and Morgan [29].

## Results and discussion

The impact of the selected factors on the individual forms of communication was assessed using multivariate statistical methods. For each form of communication, binary logistic regression was used to identify the factors that influence the use of the given form of communication in organizations. No dependence on the industry type has been found for any type of communication. Setting effective top-down communication is necessary for all types of organizations, including agricultural and forestry organizations. For the selected forms of communication, the size and type of the organization in terms of majority ownership have been identified as the significant factors for introducing the given type of communication. These determinants influence the use of communication forms, which are presented in Table 3, showing the results of tests of regression coefficients (Wald statistics) in the logistic regression model for the given communication type.

**Table 3:** Using the top-down communication types depending on the size and type of the organization.

Communication type	Statistically significant factors	Wald statistics	*p*-value
Electronic communication	The type of organization	**12.550**	**<0.001**
Communication through employee representatives or the trade union body	The type of organization	**13.315**	**<0.001**
Online meetings, webinars, video conferences	The size of organization	**14.550**	**<0.001**
Written communication, intended directly for employees	The size of organization	**14.892**	**<0.001**

Source: own survey

The following relationships characterizing the influence of the selected variables on the implementation of the given communication type have been derived from the values of the regression coefficients of the individual logistic regression models.

▪ foreign organizations are approximately three times (3.05 times) more likely to use electronic communication compared to domestic organizations;
▪ foreign organizations are more than three times (3.279 times) more likely to communicate through an employee representative or a trade union body compared to domestic ones;
▪ larger organizations (by the number of employees) are more inclined to use communication forms such as online meetings, webinars, and video conferences: specifically, organizations employing between 51 and 250 employees are approximately two times (2.027 times) more apt to use these forms than organizations employing under 50 employees; organizations employing 250 employees or more have an almost five times (4.881 times) higher probability than those employing under 50 employees;
▪ larger organizations (by the number of employees) are more likely to use communication forms of “written communication intended directly for employees”, specifically, organizations employing between 51 and 250 employees have almost the same chances as organizations employing under 50 employees (only 1.091 times higher); organizations employing 250 employees or more have more than three times (3.515 times) higher chances of using them than those employing under 50 employees.

The selected identification factors also impact the number of communication forms used by the organization. Using the logistic regression model, which compared organizations using more than three communication forms with other companies (using a maximum of three communication forms), it has been found that the number of communication forms is mostly determined by the size of the organization in terms of the number of employees. As the number of employees grows, the chance of using more than three forms of communication increases, specifically, organizations with 51–250 employees are roughly 1.4 times more likely to use more than three forms of communication compared to organizations with 50 or fewer employees; organizations with 250 and more employees are 4.77 times more likely to use more than three forms of communication than organizations with 50 or fewer employees.

A total of 30 organizations (16%) reported using only one form of communication. The majority of these organizations stated that they only use “Oral communication intended directly for employees” (19 organizations, 63%). The organizations that use only one form of communication are mostly domestic organizations (77%), smaller organizations with 50 employees or fewer (60%) and a turnover of up to 10 million euros (63%). Based on the logistic regression model for groups of organizations with only one type of communication against more than one type of communication, it can then be calculated that organizations with 51–249 employees are three times more likely to use more than one form of communication compared to those with 50 or fewer employees; organizations with 250 and more employees are almost 12 times more likely to use more than one form of communication than are those with 50 or fewer employees. On the whole, one can conclude that larger and foreign organizations use more forms of communication than organizations with Czech majority ownership.

**Fig 1:**
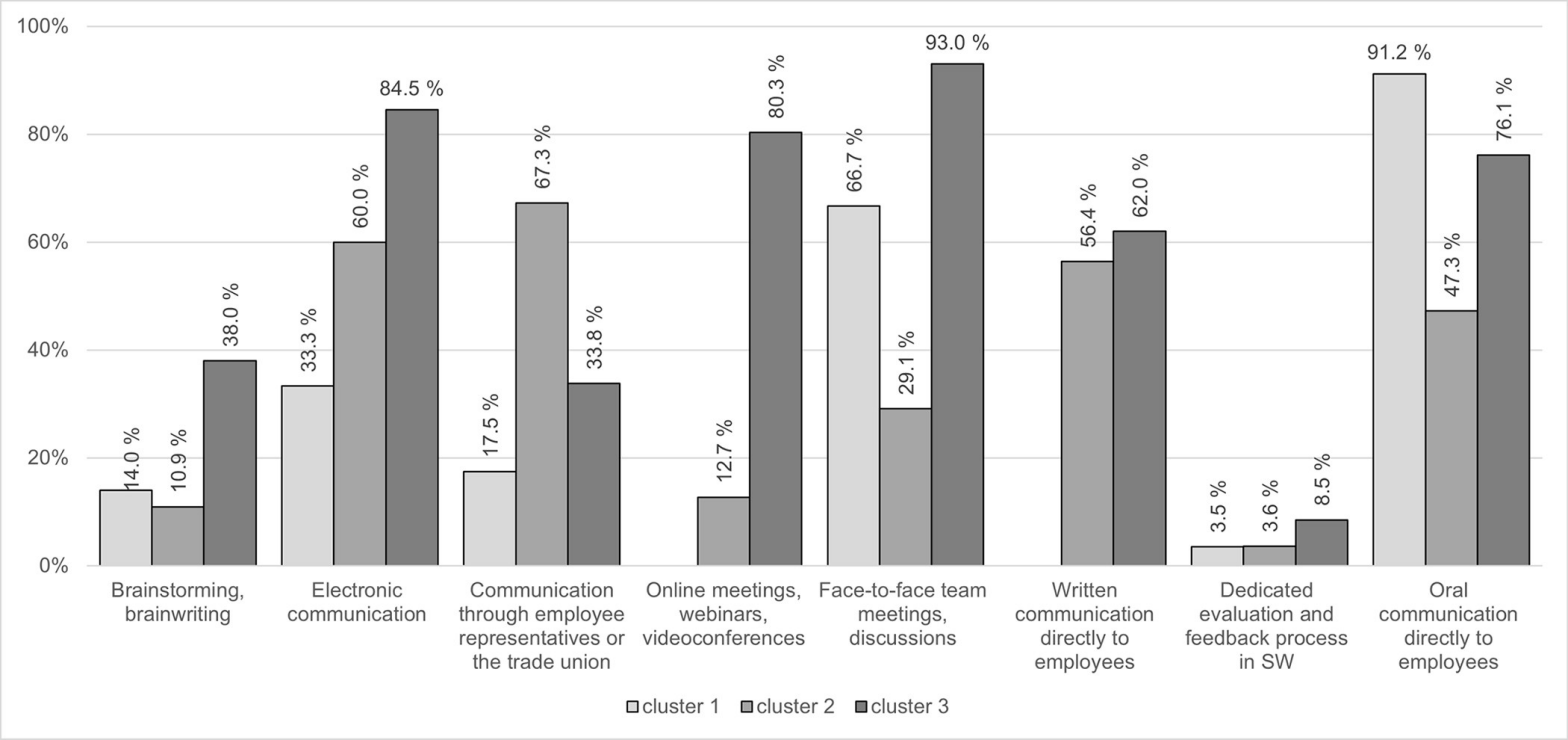
Forms of top-down communication for individual clusters. Source: own elaboration.

The use of individual forms of communication was also assessed by cluster analysis, which enabled the identification of organizations with a similar profile in terms of the established forms of top-down communication. Based on the hierarchical cluster analysis, three groups of organizations were considered. Fig 1 shows the proportion of organizations using the given type of top-down communication for individual clusters.

As Fig 1 shows, verbal communication intended directly for employees completely predominates in the first cluster (*n* = 57); it was reported by 91% of organizations in this group. Face-to-face meetings are then the second most common form of communication. None of the organizations in this group mentioned written top-down communication and online meetings or video conferences. More than 80% of the organizations in this cluster use a maximum of three forms of communication. Domestic organizations (72%) with a smaller number of employees (only 21% of organizations with 250 or more employees, 44% of organizations with a maximum of 50 employees) and a turnover under 10 million euros (53%) predominate in this group. Also, organizations from the tertiary sector predominate (58%).

On the contrary, the second cluster (*n* = 55) is characterized by rather large organizations (almost one-half of the enterprises with 250 or more employees) with foreign ownership (62%) and mid-sized turnover (40% of organizations with a turnover of 11–50 million euros), operating in the secondary sector (55%). Again, there are not too many forms of communication represented together, more than one-third of the organizations in this group use a maximum of three communication forms. Formal types of communication take the top three positions: communication through employee representatives or a trade union body (67%), electronic communication (60%), and written communication (56%).

**Fig 2:**
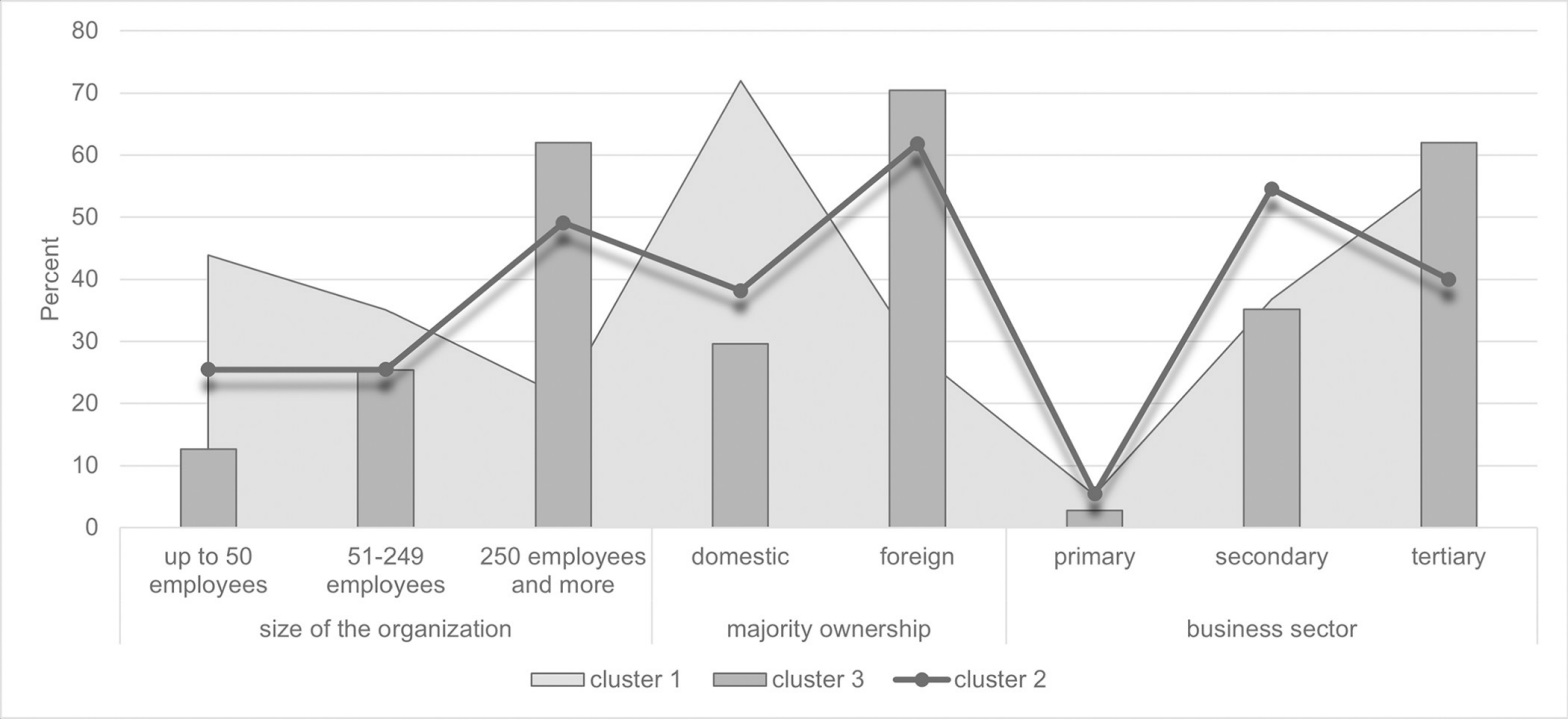
Characteristics of individual clusters based on the identification questions. Source: own elaboration.

The third cluster includes mostly large foreign organizations, which are characterized by high variability of the top-down communication forms used. Organizations from the tertiary sector predominate, with 70% of them stating foreign ownership and 62% of them having 250 or more employees. Only 16% of the organizations in this group mentioned a maximum of three forms of communication, with five forms of communication used simultaneously (39% of the organizations) being the most frequently mentioned. Forms of team communication, whether face-to-face or online, are amply represented. 93% of the organizations reported face-to-face team meetings, while 80% reported online meetings and video conferences. 85% of them reported electronic forms of communication, while oral (76%) and written (62%) communication intended directly for employees are also represented in more than half of the cases. The differences in the characteristics of individual clusters are shown in Fig 2.

Using the chi-square test of independence, it has been verified that the representation of organizations in the individual clusters is statistically significantly different in terms of the size by the number of employees (chi-square = 24.951; *p* < 0.001) and by the majority ownership (chi-square = 24.515; *p* < 0.001). There are also clear differences in terms of the core business. Given the low number of organizations in the primary sector, the difference between clusters was tested using the Fisher-Freeman-Halton Exact test, but the test result only approximates statistical significance (*p* = 0.127).

Every manager has the opportunity to influence employees through the set internal communication, but the precondition is to create a relationship of trust, which, based on the results of the focus group, can be achieved by respecting the principles that were also emphasized by Tomas Bata:

- group objectives are clearly and understandably presented to all group members without any distinction;
- all relationships are transparent and open; any problems are discussed promptly;
- group members are treated fairly;
- problems are solved immediately;
- the initiative of the group is supported;
- feedback is given to the group and feedback is also requested from the group; and
- responsibilities for specific tasks are addressed to specific people.

The importance of CSR support, including setting effective internal communication, has been growing in agricultural organizations, which is confirmed not only by the survey conducted but also by the research of Martos-Pedrero et al. [26].

It is important that every aspiring as well as experienced entrepreneur does his or her best to be able to succeed in competitive struggles that exist in every line of business which is consistent with [3, 17, 25]. Based on the evaluation of the Bata Management System towards sustainability, and not only within agricultural organizations, one can underline that an entrepreneur must become the leader of his/her employees, be their role model and at the same time an authority whom they will trust and respect. By doing so, the entrepreneur will win their trust, and the employees will embody the image of a strong organization with the ability to provide quality services to their customers and competitors. This can only be achieved on the basis of flexible and agile responses to any expected or unexpected changes or unpleasant situations in the organization, which must be urgently addressed [30]. This can be achieved, first and foremost, by setting effective top-down communication that builds respect among employees, which is in line with Rybka [25] and Vrabcová et al. [3]. Only a few studies deal with the issue of top-down communication in general, none within agriculture so far. This study is the first to be applied in agricultural organizations.

Everyone strives to be the best in their field, the primary focus when establishing any organization in any sector must be on quality resources, especially human resources, that constitute an important active element in achieving organizational goals, as evidenced by numerous studies, e.g., [1, 8, 20] and as this study points out in agricultural organizations as well.

The worldwide success of the Bata Management System was based on the training and development system, which helped to retain talented employees in the organization, for employees are the driving force of any organization, which is in line with Wang et al. [31]. These people are needed to generate innovative ideas and development through effective communication based on management conveying complete, accurate and clear information. If employees have all the necessary information and answers to their questions from the management of their organization, their loyalty is built, as confirmed by the study [13]. As the research has shown, top-down internal communication can take many forms, ranging from written and visual to electronic and oral communication, which the Bata Management System emphasized. The combination of electronic communication is supported by Brown and Lewis [32]. Plenty of organizations spend considerable amounts of money to ensure that top-down communication between management and employees, as well as between the organization and customers, is as effective, precise, fast, clear, and accurate as possible. Good communication is an essential condition for the proper functioning of an organization. Conversely, poor top-down communication results in problems that can lead to the complete bankruptcy of an organization. As current research shows, within agricultural organizations communication is not given too much emphasis and the principles of Bata are not applied. It limits the competitiveness of the management of these organizations.

As our results show, it is important to realize that, thanks to experienced and loyal employees, managers of organizations can flexibly respond to any changes in the internal or external environment and this is in accordance with Jacobs et al. 2016 [24]. The core principle of the Bata Management System, which is internationally valid, is that employees are not just machines utilised for work, but they contribute through work well-done to the desired welfare [5] of organizations and all their stakeholders [18]). The considered priority of every organization is to establish the proper strategic human resource management with modern HR trends, such as CSR, age and diversity management, or knowledge continuity management, which is stressed by the research of Vrabcová et al. [3].

Based on the analyses performed, one can conclude that the Bata Management System would have probably never been so competitive without the founder himself; his legacy has been adopted by the management of many global organizations, further developed, and become one of the most important management principles. It can be concluded that the Bata Management System, primarily the investigated importance of top-down internal communication, persists in contemporary organizations, regardless of changes in political regimes, production processes, or consumer behavior. It is still the management philosophy that strongly competes with many other management systems and has been the source of inspiration for internationally recognized organizations, e.g., Toyota in the secondary sector. The ability of personal responsibility associated with sharing in profit and loss and the desire to have as many entrepreneurial-minded employees as possible in the organization who strive to bring benefit through their work, which is perceived as Bata´s most important legacy, cannot be overlooked. To sum up, other modern organizations could undoubtedly draw inspiration from Tomas Bata´s philosophy. Such a communication setting not only can contribute to employee performance and efficiency in organizations but it is also perceived as fair and motivating by employees.

The principles of Bata management is based on the well-being of people working in organizations and it does not matter what sector we are in. The principles are also applicable in agriculture, due to the specificity of agriculture, they are not used. All processes involve communication, and effective communication will help increase profits, ensure employee training and development, and maintain a skilled workforce that is in short supply in agriculture.

We can summarize that the article fills the identified knowledge gaps summed up in the theoretical background by having presented new results in comparison across sectors and different organizations collectively. Only a primary survey in organizations can answer the question regarding the effective use of communication methods within different organizations in the past several years and set up the usage of crucial sources to achieve sustainable communication according to the Bata Management System.

In response to research question 1: For ensuring sustainable communication in accordance with the Bata Management System in organizations, it is important to combine two or more communication methods and respect the principles above.

The Bata management system persists, it is applied by production organizations in the Czech Republic and abroad, it has never been addressed in agricultural organizations, the primary sector, not even now, when sustainability, on which the Bata principle is based, is a priority in agriculture.

## Conclusion

The results have shown that combining the methods of top-down communication is crucial for effective information flow, employee stimulation, and achieving organizational goals. The principles and philosophy of the Bata Management System are based on fair and timely communication, which is even easier to implement in the present digital age than before.

The study has demonstrated that, as the number of employees grows, the chances of using more than three forms of top-down communication to convey the necessary information to everyone increases. The study results have not proven the industry’s dependence on any type of top-down communication. It has been found that the likelihood of using more than three forms of communication increases as the number of employees rises. Larger and foreign organizations use more forms of communication than those with Czech majority ownership.

The relatively small sample size is a limitation of the article; future research will focus on assessing bottom-up communication, i.e., feedback from employees towards management. The number of respondents satisfies the minimum number according to the principles of Krejcie and Morgan [29] and it can be concluded that the results may be generalized to organizations in the Czech Republic. The focus group method enhances the quality of the quantitative research and complements the quantitative research with the specifics of the agricultural sector.

This study has several limitations. In particular, it focuses only on organizations in the Czech Republic and the results cannot be generalized to the entire population; it is, an important sample of organizations across sectors and sizes. Research in organizations in the Czech Republic is justifiable, as Tomas Bata was a Czechoslovak manager whose influence was worldwide. The results are valuable for every manager looking for a way to set effective sustainable top-bottom communication. The results emphasize the managerial role in the setting of internal knowledge and being able to keep and use it appropriately. We are fully aware that the study has theoretical and methodological limitations that may affect the validity of this study’s findings. It can be considered a limitation of the research that the results come from the data and answers provided by the representatives of the companies in the questionnaire survey. It is necessary to interpret the observations in the context of the aforementioned research sample. Respondents may have tended to create an enhanced business image and appear unduly rational. Nevertheless, the questions were asked in a non-leading manner and in compliance with the rules of social science research. The research results presented in Web of Science until 2022 were analysed by checking the research on the Google Scholar citation tracker to reduce these limitations.

According to the available research results presented in the Web of Science, no research was conducted on the importance of top-down communication from above when applying the principles of Bata in the conditions of agricultural enterprises. The article was contributed by advancing knowledge of the principles of Bata with a focus on effective communication in agricultural organizations. The application of these principles in agricultural organizations will help the development of the field and the solution of managerial problems caused by ineffective communication causing conflicts or problems in following processes.

## Supporting information

S1 File (CSV)
